# Targeting advanced glycation end products: potential therapeutic approaches for mitigating diabetic intervertebral disc degeneration?

**DOI:** 10.3389/fendo.2025.1618984

**Published:** 2025-07-07

**Authors:** Luyang Wang, Qipeng Shao, Haiyang Wu, Cheng Li

**Affiliations:** ^1^ Department of Orthopaedics, The First Affiliated Hospital of Zhengzhou University, Zhengzhou, China; ^2^ Department of Orthopaedics, Ganzhou People’s Hospital, Ganzhou, China; ^3^ Department of Spine Surgery, Wangjing Hospital, China Academy of Chinese Medical Sciences, Beijing, China; ^4^ Center for Musculoskeletal Surgery (CMSC), Charité-Universitätsmedizin Berlin, corporate member of Freie Universität Berlin, Humboldt University of Berlin, and Berlin Institute of Health, Berlin, Germany

**Keywords:** advanced glycation end products, diabetes mellitus, intervertebral disc degeneration, therapeutic target, AGEs

## Abstract

Diabetes mellitus is strongly associated with accelerated intervertebral disc degeneration, a condition that significantly contributes to lower back pain and reduced quality of life. Emerging evidence indicates that advanced glycation end products (AGEs) are key mediators in the pathophysiology of disc degeneration through the stimulation of inflammatory pathways, promotion of oxidative stress, and induction of extracellular matrix modifications. This review critically examines current literature on the role of AGEs in diabetic disc degeneration and evaluates potential therapeutic interventions aimed at mitigating these deleterious effects. Targeting AGEs represents a promising therapeutic avenue to mitigate diabetic intervertebral disc degeneration. The current evidence supports the rationale for further investigation into AGE inhibitors, cross-link breakers, and receptor for AGEs modulators as potential treatment strategies. However, to translate these findings into clinical practice, well-designed clinical trials are required to validate the efficacy and safety of these interventions, as well as to optimize treatment protocols.

## Introduction

Amidst rapid global economic development and concomitant lifestyle transformations, modern society has witnessed the proliferation of dietary patterns characterized by excessive fat and sugar consumption, coupled with sedentary behaviors (1). These maladaptive trends have precipitated widespread metabolic dysregulation, including insulin resistance and pancreatic β-cell dysfunction, thereby fueling the diabetes pandemic (2). Substantial clinical evidence demonstrates that diabetes mellitus (DM) not only predisposes individuals to cardiovascular diseases, nephropathy, and neuropathies but also exerts profound detrimental effects on spinal integrity and function (3). Notably, diabetes-induced intervertebral disc degeneration (DIDD), often overlooked due to its insidious onset and progressive nature, significantly compromises patients’ quality of life through chronic low back pain and functional impairment (4, 5). Furthermore, persistent hyperglycemia exacerbates systemic oxidative stress and low-grade inflammation, both of which accelerate degenerative cascades within disc tissues (6). IDD in diabetic patients complicates therapeutic interventions, prolongs rehabilitation, and escalates healthcare expenditures, underscoring the imperative to elucidate the pathomechanisms linking these conditions and identify novel therapeutic avenues.

Recent scientific inquiry has increasingly focused on the instrumental role of advanced glycation end products (AGEs) in this pathological nexus. AGEs arise through non-enzymatic glycation reactions between reducing sugars and proteins, lipids, or nucleic acids, culminating in complex cross-linking and structural modifications (7). Their formation is dynamically regulated by glycemic levels, oxidative stress, and pro-inflammatory milieus (8). While AGE generation proceeds gradually under physiological conditions, diabetic hyperglycemia and oxidative stress synergistically amplify their accumulation (9). Ligand engagement with the receptor for AGEs (RAGE) activates downstream signaling cascades, triggering robust pro-inflammatory cytokine release and oxidative damage, which collectively drive tissue injury and functional decline (10). Moreover, AGE deposition in vascular, neural, and renal tissues is intimately associated with diabetic complications. Such pathological accrual not only induces cellular damage but also disrupts extracellular matrix architecture by promoting aberrant collagen cross-linking, thereby impairing tissue biomechanics and biological homeostasis (11). Against this backdrop, investigations into AGE-mediated organ pathology have intensified, with DIDD, a multifactorial chronic degenerative disorder, emerging as a critical area of research.

The intervertebral disc (IVD), comprising the nucleus pulposus (NP), annulus fibrosus (AF), and cartilaginous endplates (CEP) (12), serves as a keystone structure in maintaining spinal stability and mobility (13). However, aging superimposed with metabolic perturbations precipitates functional decline in disc cells and matrix disorganization, ultimately culminating in degenerative changes. In diabetes, chronic hyperglycemia drives excessive AGE formation (14), which not only hastens damage to vascular and neural systems but also disrupts disc cell metabolism and extracellular matrix remodeling (15). Consequently, deciphering the mechanistic contributions of AGEs to DIDD holds substantial promise for unraveling disease pathogenesis and revealing targeted therapeutic strategies.

## Formation of AGEs and their pathological significance in DM

The formation of AGEs predominantly stems from the Maillard reaction, wherein reducing sugars react with biological macromolecules under hyperglycemic conditions, ultimately yielding irreversible terminal products through complex intermediate stages (16, 17). Hyperglycemia not only accelerates the kinetics of this reaction (18) but also, under oxidative stress, facilitates the oxidation of intermediate compounds into stable AGEs (19). These AGEs subsequently engage in covalent cross-linking with target macromolecules, thereby altering their physicochemical properties (20). In diabetic patients, AGE formation and accumulation significantly exceed physiological levels observed in healthy individuals (21). They are not only detectable in systemic circulation but also deposit extensively within the extracellular matrix of tissues, disrupting collagen cross-linking and compromising biomechanical integrity (22).

Notably, AGEs originate from both endogenous synthesis and exogenous dietary sources, collectively exacerbating tissue AGE burden (23). Their deleterious effects on organ systems are primarily mediated through binding to cell-surface receptors, most notably the receptor for AGEs (RAGE). The engagement of AGEs with RAGE triggers the activation of multiple intracellular signaling cascades-including NF-κB, MAPK, JNK, and TGF-β pathways-culminating in inflammatory responses, cellular stress, and apoptosis (24). This activation not only stimulates excessive production of cytokines and chemokines but also induces reactive oxygen species (ROS) generation, establishing a self-perpetuating cycle that amplifies tissue damage.

The AGE-RAGE axis serves as a critical molecular “trigger” for downstream pathological events. For instance, in renal pathophysiology, AGE-RAGE signaling upregulates fibrogenic mediators such as TGF-β and connective tissue growth factor (CTGF), directly promoting tubulointerstitial fibrosis-a hallmark of diabetic nephropathy (25). These mechanistic insights provide a robust molecular foundation for therapeutic strategies targeting the AGE-RAGE pathway, offering potential avenues for mitigating DIDD.

## The interrelationship between DM and IDD

IDD arises from a multifactorial etiology involving age-related changes, mechanical stress, and increasingly recognized metabolic dysregulation (26). Recent clinical and basic research has consistently demonstrated that diabetic patients exhibit accelerated IDD progression and more severe degenerative changes compared to non-diabetic individuals (27). To elucidate the contributory roles of various factors, large-scale cohort studies employing advanced imaging modalities such as magnetic resonance imaging (MRI) have systematically evaluated IDD incidence and progression across populations. A seminal 4-year longitudinal study by Teraguchi et al. tracked 617 Japanese participants, analyzing lumbar MRI data to assess IDD progression, incidence rates, and risk factors. The findings revealed universal disc degeneration among type 2 diabetes mellitus (T2DM) patients, with a statistically significant positive correlation between T2DM and degeneration in upper lumbar segments (L1/2-L3/4), establishing T2DM as an independent risk factor for IDD in these regions (28). Complementing these results, a retrospective Chinese study of 772 chronic low back pain patients (622 with T2DM) stratified participants by glycemic control (optimal vs. suboptimal) and disease duration (≤10 vs. >10 years). Patients with >10-year T2DM duration and poor glycemic control exhibited markedly greater IDD severity than other subgroups. Moreover, degeneration severity at all lumbar levels (L1/2–L5/S1) strongly correlated with T2DM duration (29). These findings robustly validate T2DM’s pivotal role in IDD pathogenesis while implicating prolonged disease duration and inadequate glycemic control as key accelerants of degenerative progression (30).

From a biomechanical perspective, T2DM induces detrimental changes in disc structure and material properties. Experimental studies demonstrate that hyperglycemia disrupts collagen fiber organization in the annulus fibrosus while increasing non-enzymatic cross-linking, thereby compromising resistance to compressive and shear stresses (31). This mechanical weakening predisposes discs to injury from physiological loads, hastening degeneration. Concurrently, chronic hyperglycemia alters disc hydration and proteoglycan content, further impairing shock-absorbing capacity and spinal stability (32).

Genetic predisposition plays a non-negligible role in IDD pathogenesis, as evidenced by twin studies and genomic analyses. Notably, the metabolic disturbances characteristic of T2DM may potentiate this genetic vulnerability, rendering high-risk individuals more susceptible to severe disc degeneration (30). Thus, the co-occurrence of T2DM and IDD across populations likely represents a convergence of genetic, environmental, and metabolic insults, a paradigm that underscores the need for personalized therapeutic approaches.

## Potential mechanistic role of AGEs in DIDD

The IVD is anatomically composed of three distinct structural components: the outer AF, the central NP, and the superior/inferior CEP. Under chronic hyperglycemic conditions characteristic of DM, the progressive accumulation of AGEs exerts deleterious effects on both the structural integrity and biological function of these disc components through the following pathomechanisms. AGEs significantly affect the function and stability of these structures through mechanisms such as oxidative stress, inflammatory responses, and extracellular matrix (ECM) degradation. Although these structures are affected in different ways, the mechanisms of action of AGEs share commonalities. This article will explore these commonalities and differences in detail.

### Annulus fibrosus

Functioning as the principal load-bearing structure that maintains disc stability under complex biomechanical stresses, the AF exhibits a sophisticated multilamellar architecture with unique histological characteristics (33). Contemporary morphological studies reveal that the AF comprises 15–25 concentric lamellae with individual thickness ranging from 0.14-0.52 mm, wherein collagen fibers adopt a precise crisscross arrangement that confers exceptional tensile strength and shear resistance (34). Biochemically, the AF extracellular matrix consists of approximately 20% proteoglycans and 60% collagen, with type I collagen fibers organized in concentric lamellae predominating (35, 36). This highly specialized architecture enables the AF to effectively counteract circumferential stresses transmitted from the NP while maintaining overall disc stability (37). However, AGE accumulation can disrupt this specialized structure, impairing the AF’s ability to handle mechanical loads.

Anatomically, the AF can be subdivided into outer and inner regions (38). The outer AF predominantly contains type I collagen fibers with superior tensile properties, while the inner AF serves as a transitional zone to the NP, exhibiting reduced cellular density and less organized matrix composition (39). The mechanical competence of the AF critically depends on both the structural integrity of collagen fibers and their precise spatial orientation. The alternating angular arrangement of collagen lamellae provides optimal resistance against radial stresses from the NP, thereby preventing disc displacement and structural collapse (40).

In the previous sections, we discussed in detail the structural characteristics of the AF and the precise arrangement of collagen fibers. However, excessive AGE accumulation within the IVD induces detrimental structural modifications through pathological collagen crosslinking, which not only disrupts the native fiber organization but also promotes aberrant crosslink formation, ultimately compromising the AF’s adaptive capacity to mechanical loading (41). These molecular and ultrastructural alterations collectively contribute to AF dysfunction and progressive disc degeneration.

Advances in modern imaging modalities have provided unprecedented insights into disc degeneration mechanisms. Utilizing two-photon imaging technology, researchers have demonstrated that high-AGE diets significantly exacerbate collagen fiber damage within the AF, with particularly pronounced effects observed in female specimens (42). This finding provides a new perspective on how AGEs affect the structure of the intervertebral disc at the macroscopic level. Based on these imaging observations, subsequent studies have further revealed the pathological mechanisms associated with AGEs, particularly their specific effects on the annulus fibrosus at the cellular level.

Based on these imaging findings, Experimental evidence indicates that AGEs potently suppress AF cell proliferation while promoting apoptotic cell death (40). At the molecular level, AGEs modulate apoptotic pathways through upregulation of pro-apoptotic Bax, downregulation of anti-apoptotic Bcl-2, and subsequent cytochrome c release from mitochondria, thereby activating caspase-9 and caspase-3 mediated apoptosis cascades. Concurrently, AGEs induce mitochondrial dysfunction characterized by elevated ROS production and diminished membrane potential, further exacerbating cellular apoptosis (43). Moreover, AGEs compromise the mechanical stability of the AF by disrupting physiological collagen crosslinking patterns (44). Through these dual mechanisms - inducing cellular apoptosis and impairing matrix structural integrity AGEs synergistically accelerate disc degeneration at both structural and functional levels in the diabetic context.

### Nucleus pulposus

The effects of AGEs on the intervertebral disc are not limited to the annulus fibrosus, but also involve the NP. The NP, as the central region of the intervertebral disc, is composed of highly functional NP cells and their secreted extracellular matrix (ECM). NP cells are not only responsible for synthesizing and maintaining the ECM, which is rich in proteoglycans, type II collagen, and hyaluronic acid (45), but also play a decisive role in preserving the disc’s osmotic properties, elasticity, and biomechanical homeostasis (46).

Numerous studies have demonstrated that the progressive accumulation of AGEs in intervertebral disc tissues can cause direct damage and functional impairment to NP cells through multiple molecular mechanisms (47). First, AGEs reduce NP cell viability, suppress proliferation, and induce apoptosis, thereby decreasing cell numbers (48). Furthermore, under diabetic conditions, AGE accumulation not only triggers NP cell apoptosis but also diminishes ECM synthesis capacity while upregulating the expression of ECM-degrading enzymes such as MMP-13, ultimately leading to significant ECM degeneration (49). This process is not only associated with the direct structural damage to proteins by AGEs but is also closely linked to the aberrant activation of intracellular signaling pathways. For instance, AGEs can activate the receptor for AGEs (RAGE), triggering NF-κB and MAPK signaling pathways, which subsequently induce the secretion of pro-inflammatory cytokines and matrix-degrading enzymes, further exacerbating ECM destruction (50, 51).

In addition to directly impairing NP cell function, AGEs also induce disordered intermolecular crosslinking by covalently modifying extracellular proteins, particularly long-lived structural proteins such as collagen and elastin, leading to tissue stiffening and dysfunction. This crosslinking not only alters the mechanical properties of the ECM but also promotes the secretion of inflammatory cytokines and matrix metalloproteinases (MMPs) through the activation of intracellular signaling pathways like NF-κB and MAPK, thereby accelerating ECM degradation (50). Previous studies have identified IL-1β and TNF-α as the most critical pro-inflammatory mediators in disc pathology, as they induce apoptosis, accelerate ECM degradation, and disrupt disc integrity by upregulating matrix-degrading enzymes such as MMPs and ADAMTS (52). Concurrently, serum levels of pro-inflammatory cytokines (e.g., IL-6) correlate positively with clinical symptom severity, further underscoring the pivotal role of these cytokines in disc degeneration and associated pain pathogenesis (10).

In the previous sections, we discussed how AGEs influence the mechanical stability of the intervertebral disc and accelerate disc degeneration by altering the structure of the annulus fibrosus and nucleus pulposus. AGEs not only change the physical properties of the matrix through pathological collagen crosslinking but also further exacerbate disc degeneration by activating inflammatory responses. Experimental studies have shown that direct injection of AGEs into mouse intervertebral discs significantly increases the expression of the pro-inflammatory cytokine IL-23 while reducing levels of the protective anti-inflammatory cytokine IL-10, further confirming AGEs’ involvement in disc inflammation (53). Moreover, AGE-induced upregulation of TNF-α can promote nerve fiber ingrowth into degenerative discs, exacerbating pain symptoms, as evidenced clinically by imaging findings such as the “black disc” phenomenon on T2-weighted MRI (52). This pro-inflammatory-mediated pathological process explains why diabetic patients are more prone to painful disc herniation during disc degeneration.

Additionally, AGEs exacerbate intracellular oxidative stress by inducing ROS production (42), which not only disrupts intracellular signaling networks but also activates processes such as FAM134B-mediated endoplasmic reticulum (ER) phagocytosis, further compromising organelle homeostasis (54). The ER, a critical site for protein synthesis, folding, and quality control, is essential for cell survival (55). When ER stress occurs, misfolded or unfolded proteins accumulate, triggering the unfolded protein response (UPR) (56). Under moderate stress, the UPR serves as a cytoprotective mechanism to restore homeostasis; however, under prolonged or severe stress, it initiates apoptotic pathways (57).

The endoplasmic reticulum is the main storage site of calcium ions within the cell and is crucial for maintaining calcium homeostasis. Research indicates that AGE treatment of NP cells leads to sustained cytoplasmic Ca^2+^ elevation and ER Ca^2+^ depletion, disrupting calcium homeostasis and contributing to ER stress (58). Furthermore, AGEs significantly affect the activity of ER Ca^2+^ channels, including inositol 1,4,5-trisphosphate receptors (IP3Rs), ryanodine receptors (RyRs), and the sarco/endoplasmic reticulum Ca^2+^-ATPase (SERCA) pump, with their dysfunction directly participating in AGE-induced NP cell pathology (59). In NP cells, ER stress is a double-edged sword: it can initiate protective mechanisms but may also induce apoptosis under excessive stress. AGEs activate key transmembrane proteins in the ER, initiating the UPR, while downstream effectors such as C/EBP homologous protein (CHOP) regulate pro-apoptotic gene expression, driving cells toward programmed death under severe or persistent stress (60).

Moreover, AGEs not only disrupt intracellular redox balance by promoting ROS generation but also impair mitochondrial membrane permeability, increasing pro-apoptotic Bax levels while decreasing anti-apoptotic Bcl-2 (61). This imbalance further elevates intracellular ROS levels and prolongs mitochondrial permeability transition pore (mPTP) activation, accelerating apoptosis.

### Cartilage endplate

The effects of AGEs on the intervertebral disc are not limited to the annulus fibrosus and nucleus pulposus, but also involve the CEP, which is another critical component in disc degeneration. The CEP serves as a critical bridge connecting the vertebral body and intervertebral disc, not only facilitating the transport of essential nutrients to the disc but also maintaining its internal homeostasis. Its biomechanical function and barrier properties are vital for overall disc health (27, 62). However, both clinical and experimental evidence indicate that the disruption of CEP integrity and function often acts as an “initiator” of further disc degeneration. Such damage not only impairs nutrient exchange but also activates local inflammatory responses and matrix degradation pathways, accelerating the degenerative process of the entire disc.

Due to its high collagen and proteoglycan content and long half-life, the CEP is particularly susceptible to the accumulation of AGEs (63). AGE deposition may induce abnormal cross-linking between collagen molecules, altering the mechanical properties of the CEP, reducing its permeability and elasticity, and ultimately compromising nutrient diffusion within the disc (44). Furthermore, AGEs binding to the receptor for AGEs (RAGE) on CEP cells can trigger localized inflammation, promoting the secretion of matrix-degrading enzymes such as MMPs, thereby accelerating ECM degradation in both the CEP and the entire disc (64).

Additionally, numerous animal models and clinical studies have demonstrated that disc degeneration progresses more rapidly in diabetic conditions. Experimental data from diabetic rat models reveal significant reductions in disc matrix content, increased apoptosis, and structural deterioration of the CEP (65). However, most existing research has focused on the degeneration mechanisms of the nucleus pulposus and annulus fibrosus, while studies on structural and functional alterations of the CEP under diabetic conditions remain relatively scarce (66). This gap limits a comprehensive understanding of the pathophysiological mechanisms underlying disc degeneration.

In recent years, advances in molecular biology and imaging techniques have drawn increasing attention to the role of the CEP in disc degeneration. The CEP is not only a crucial pathway for disc nutrition but also determines the biomechanical properties and disease resistance of the disc through its unique matrix and cellular composition (62, 67). In diabetic patients, chronic hyperglycemia leads to AGE deposition in the CEP, which may induce collagen cross-linking, matrix stiffening, and functional impairment of CEP cells, triggering local inflammation and oxidative stress. These changes further weaken the CEP’s barrier function, reduce nutrient supply to the disc, and promote overall disc degeneration (27).

Moreover, AGE-stimulated CEP cells may undergo apoptosis or functional abnormalities, and their secreted regulatory factors can disrupt the metabolic balance of the ECM in the microenvironment, contributing significantly to disc degeneration (68, 69). Beyond the direct molecular pathological mechanisms mediated by AGEs, growing evidence suggests that systemic metabolic dysregulation in diabetes exacerbates disc degeneration through chronic inflammation and oxidative stress. Prolonged glycemic fluctuations and insulin resistance induce a low-grade systemic inflammatory state, which not only damages vascular endothelium but also disrupts the metabolic equilibrium of CEP cells (53). Furthermore, systemic oxidative stress enhances the interaction between AGEs and RAGE, creating a vicious cycle that further diminishes the damage resistance of the CEP and the entire disc (66).

## Common mechanisms

The mechanistic role of AGEs in the IVD exhibits commonality across different structural components, including the AF, NP, and CEP. These common mechanisms primarily involve oxidative stress, ER stress, collagen crosslinking and matrix stiffening, and inflammatory responses. AGEs promote the generation of ROS, inducing intracellular oxidative stress that disrupts cellular function and triggers pathological changes. This process plays a significant role in AF, NP, and CEP, impairing normal cellular activities and accelerating disc degeneration. Simultaneously, AGEs initiate ER stress, leading to calcium imbalance and the UPR, which induces cell apoptosis. This reaction is evident in all regions, directly damaging cell function and promoting cell death through the activation of pro-apoptotic genes, further exacerbating disc degeneration. Additionally, AGEs induce abnormal crosslinking between collagen molecules, altering the mechanical properties of the matrix, leading to tissue stiffening and dysfunction, thereby weakening the structural stability of the disc and affecting its compressive resistance and elasticity. AGEs also activate local inflammation, promoting the secretion of matrix-degrading enzymes such as MMPs, which further accelerates ECM degradation. By binding to the RAGE, AGEs activate inflammatory signaling pathways such as NF-κB and MAPK, enhancing the release of pro-inflammatory cytokines and matrix-degrading enzymes, thereby accelerating disc degeneration. In conclusion, AGEs act through multiple mechanisms in AF, NP, and CEP, broadly impacting the structure and function of the intervertebral disc, ultimately accelerating the progression of degenerative disc disease.

## Targeting AGEs for DIDD treatment strategies

In-depth research into the role of AGEs in DIDD not only helps clarify the fundamental pathological processes of the disease but also provides new insights for identifying effective clinical treatment interventions. Given the central role of AGEs and their interaction with the RAGE axis in DIDD pathogenesis, therapeutic strategies targeting this pathological pathway have attracted widespread attention in recent years. These treatment approaches can be mainly categorized into the following aspects:

### Inhibiting AGEs formation

Inhibiting AGEs formation is one of the most direct intervention methods, with its basic principle being to block the non-enzymatic glycation reaction between reducing sugars and proteins, thereby reducing AGEs production. In clinical and experimental studies, AGE inhibitors such as aminoguanidine and pyridoxamine have been extensively researched and shown good intervention effects (70, 71). Experimental data indicate that these inhibitors can significantly reduce AGEs formation, thereby decreasing oxidative stress and inflammatory responses induced by AGEs accumulation (71). Additionally, some natural polyphenolic compounds exhibit similar AGE-inhibitory effects, possessing both antioxidant capabilities and the ability to trap reactive intermediates to block AGEs generation (72). These studies provide strong theoretical support and experimental evidence for the application of AGEs inhibition in DIDD treatment.

### Blocking AGEs-RAGE interaction

Another important therapeutic strategy involves blocking the interaction between AGEs and RAGE to prevent the activation of downstream pathological signals. Currently, approaches such as RAGE monoclonal antibodies, soluble RAGE (sRAGE), and RNA interference-based technologies have achieved certain success in animal models (73, 74). For example, anti-RAGE monoclonal antibodies can significantly reduce the expression of inflammatory and fibrotic factors in endothelial cells, thereby improving organ function (74). Furthermore, researchers have developed nanoparticle-based siRNA delivery systems to specifically knock down RAGE expression, providing a novel approach for precise regulation of AGE-RAGE signaling in clinical settings (75).

### Clearing AGEs

Clearing already-formed AGEs, known as the “AGEs breaker” strategy, has also become a research hotspot in recent years. AGEs breakers degrade AGEs-induced crosslinks, thereby improving the biomechanical properties of proteins and organ function. AGE crosslink breakers such as N-phenacylthiazolium bromide (PTB) and ALT-711 have been shown in animal experiments to partially reverse tissue stiffness and dysfunction caused by AGEs accumulation (76). This approach has demonstrated clear benefits not only in the cardiovascular system but also shows potential therapeutic value in diabetic retinopathy and nephropathy (73). However, due to the diversity and widespread distribution of AGEs, the clinical translation of such drugs still faces many challenges, requiring further optimization of their pharmacokinetics and safety profiles.

### Other treatment strategies and prospects for combined applications

Beyond the aforementioned strategies directly targeting AGEs and RAGE, antioxidants, anti-inflammatory drugs, and stem cell therapy have gradually shown potential in DIDD intervention. Since AGEs exert their pathogenic effects by activating oxidative stress and inflammatory pathways, the use of antioxidants (e.g., N-acetylcysteine) can interrupt this pathological vicious cycle (77). Additionally, non-steroidal anti-inflammatory drugs (NSAIDs) and drugs targeting the NF-κB signaling pathway can effectively reduce AGEs-RAGE-mediated inflammatory responses, thereby improving organ function (78). Stem cell therapy, as an important direction in regenerative medicine, has also garnered increasing attention for its application in repairing AGEs-induced cell damage and tissue degeneration, providing new insights into the synergistic effects of multiple treatment strategies.

From an overall perspective, single treatment modalities are insufficient to completely block or reverse the complex role of AGEs in DIDD pathogenesis. Therefore, multi-target combination therapy has become an important research trend. For example, combining AGEs inhibitors with antioxidants can reduce AGEs generation while alleviating oxidative stress. Meanwhile, the combined application of RAGE blockers and AGE breakers may simultaneously prevent new AGEs signaling and clear existing pathological crosslinks (79). Furthermore, natural plant extracts, with their multi-target and low-toxicity characteristics, show great potential in combination therapy strategies (72).

## Future perspectives

Although recent years have seen abundant literature reporting on the role of AGEs in diabetic complications, many unanswered questions remain regarding the intrinsic relationship between AGEs and DIDD. First, current research on DIDD pathological mechanisms primarily focuses on isolated processes such as apoptosis, matrix degradation, and oxidative stress, while the fine regulatory mechanisms by which AGEs synergistically induce cellular dysfunction through multiple signaling pathways remain incompletely elucidated (53). Second, most experimental data come from *in vitro* cell models or animal models, with limited direct evidence on human DIDD pathogenesis, making the translation from laboratory findings to clinical applications challenging (80). Additionally, most studies concentrate on AGEs’ functional changes in single cell types (e.g., nucleus pulposus cells or annulus fibrosus cells), whereas the actual intervertebral disc, as a complex multi-cellular, multi-matrix structure, has not been sufficiently explored in terms of its intrinsic intercellular interactions and cross-regional signaling mechanisms (69). Moreover, the expression and activation of AGEs-RAGE signaling pathways may vary among different patient populations, and how to develop precise interventions for such individual differences remains an urgent issue for future research (81). Furthermore, current treatment strategies mostly focus on blood glucose control or AGEs inhibitors, but their efficacy in improving DIDD is suboptimal. Therefore, there is an urgent need to develop more targeted interventions, such as using molecular nanotechnology to directly clear or degrade AGEs within intervertebral discs.

In summary, current research indicates that AGEs play a central role in diabetic complications. Their abnormal deposition not only triggers local oxidative stress and pro-inflammatory responses but also mediates apoptosis and structural damage through binding with RAGE, thereby accelerating disc degeneration. Based on this understanding, multi-level therapeutic strategies targeting AGEs, including inhibiting AGEs formation, blocking AGEs-RAGE interactions, and clearing existing AGEs, provide novel insights for the prevention and treatment of DIDD. Meanwhile, exploring multi-target combination therapies by integrating antioxidants, anti-inflammatory agents, and stem cell approaches may further enhance treatment efficacy and improve patient outcomes.
